# C-phycocyanin protects against low fertility by inhibiting reactive oxygen species in aging mice

**DOI:** 10.18632/oncotarget.8165

**Published:** 2016-03-17

**Authors:** Yan-Jiao Li, Zhe Han, Lei Ge, Cheng-Jie Zhou, Yue-Fang Zhao, Dong-Hui Wang, Jing Ren, Xin-Xin Niu, Cheng-Guang Liang

**Affiliations:** ^1^ The Key Laboratory of National Education Ministry for Mammalian Reproductive Biology and Biotechnology, The Research Center for Laboratory Animal Science, College of Life Science, Inner Mongolia University, Hohhot, Inner Mongolia, People's Republic of China

**Keywords:** ovarian aging, oocyte, oxidative stress, C-phycocyanin, D-galactose, Gerotarget

## Abstract

Women over 35 have higher rates of infertility, largely due to deterioration of oocyte quality characterized by fragmentation, abnormal meiotic spindle-chromosome complexes, and oxidative stress. C-phycocyanin (PC) is a biliprotein enriched in *Spirulina platensis* that is known to possess antioxidant, anti-inflammatory, and radical-scavenging properties. D-galactose-induced aging acceleration in mice has been extensively used to study aging mechanisms and for pharmaceutical screening. In this study, adult female B6D2F/1 mice injected with D-galactose were used as a model to test the age-reversing effects of PC on degenerated reproductive ability. Our results show that PC can prevent oocyte fragmentation and aneuploidy by maintaining cytoskeletal integrity. Moreover, PC can reverse the expression of antioxidant genes, increase superoxide dismutase (SOD) activity and decrease methane dicarboxylic aldehyde (MDA) content, and normalize mitochondria distribution. PC exerts its benefit by inhibiting reactive oxygen species (ROS) production, which decreases apoptosis. Finally, we observe a significant increase in litter size after PC administration to D-galactose-induced aging mice. Our study demonstrates for the first time that D-galactose-induced impaired female reproductive capability can be partially rescued by the antioxidant effects of PC.

## INTRODUCTION

The human female reproductive system ages more rapidly than most other body systems, and reproductive capacity is negatively correlated with age [1, 2]. For a variety of reasons, many women postpone childbearing, and a considerable proportion of aged female become infertile [3]. A reduction in quantity and deterioration in oocyte quality is universal in women over 40. Poor oocyte quality is characterized by meiotic spindle anomalies, chromosome misalignment, oxidative stress, gene expression changes, shortened telomeres, and loss of cohesion [1, 4-6].

Oxidative stress occurs due to gradual accumulation of damage by free radicals that are generated during normal metabolism, and it is considered one of the major mechanisms underlying aging [7, 8]. Germ cells initiate meiosis and arrest at the dictyate stage of prophase I in the fetal ovary; these postnatal germ cells remain arrested for weeks to months in mice and 10-50 years in humans [9]. During this prolonged interval, reactive oxygen species (ROS) accumulate and decrease oocyte quality and quantity [10]. ROS negatively affect processes from oocyte maturation to fertilization, embryo development, and pregnancy, and they are associated with the age-related decline in reproduction [3, 11, 12]. Previous reports demonstrated that ROS accumulation in cells can lead to cytoskeletal derangement [13], shortened telomeres [14], impaired telomerase activity [15], antioxidant system dysfunction [16, 17], disturbances of ATP levels [18] and mitochondrial distribution [19], and cell apoptosis [20].

D-galactose (D-gal) is a reducing sugar that can form advanced glycation end products (AGEs) *in vivo*. Aging is accelerated after mice receive oral or subcutaneous D-gal [21-23]. Some studies focused on the effect of D-gal on oxidative stress, and one found that AGEs can cause the accumulation of ROS, especially superoxide radicals and hydrogen peroxide [21].

Reducing oxidative stress by antioxidant supplementation could potentially reduce ROS-induced damage, thus maintaining oocyte and follicle number and quality [24-27]. C-phycocyanin (C-Pc, PC) is a major biliprotein in *Spirulina platensis* that possesses antioxidant, neuroprotective, anti-inflammatory, and radical-scavenging properties [28-30], suggesting PC as a potential agent for preventing ROS-induced aging or ROS damage [31, 32]. However, little is known about whether PC can prevent D-gal-induced aging and impaired reproductive ability. In the present study, we treated mice with PC to investigate whether it could preserve reproductive performance in a D-gal-induced aging model.

## RESULTS

### PC reversed some organ coefficients in D-gal-induced aging mice

**Figure 1:**
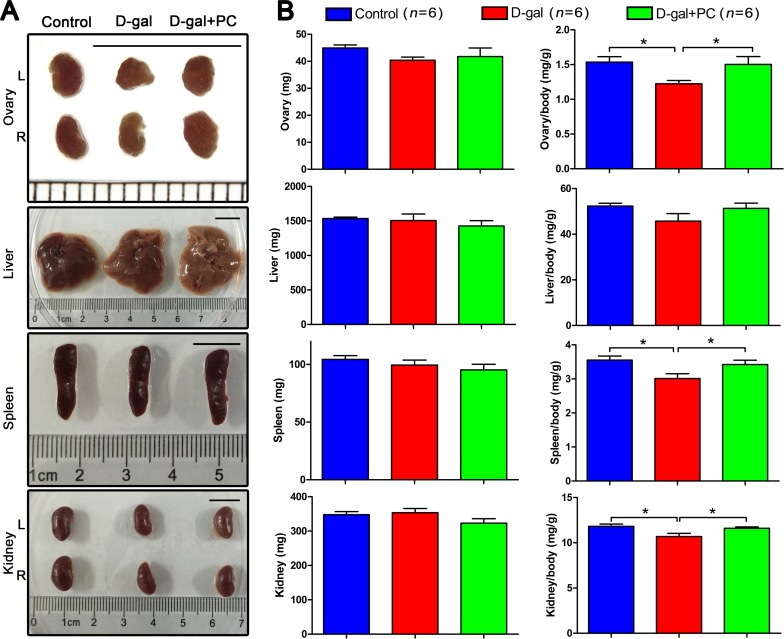
PC reversed the organ coefficients of the ovary, spleen, and kidney in D-gal-induced aging mice **A.** Organ views. No obvious abnormal morphology was observed after D-gal or PC administration. Scale bar = 1 cm. L: left, R: right. **B.** Body weight and organ coefficients. No statistical difference was observed for ovary, liver, spleen, or kidney weight. However, the organ coefficients of the ovary, spleen, and kidney from the control and D-gal+PC groups were higher than those in the D-gal group. Data are presented as means ± SEMs and were processed by one-way ANOVA and Newman-Keuls post hoc tests. Significant differences between groups, *P < 0.05. *n* indicates the number of mice for each treatment.

We first examined macroscopic views of mouse organs in the control, D-gal, and D-gal+PC groups and found no obvious morphologic changes (Figure 1A). We then compared organ weight and organ coefficients (organ weight/body weight) among the three groups. There were no statistical differences in the weights of the ovary, liver, spleen, or kidney. Similarly, there was no significant difference in the liver organ coefficient (Figure 1B, Table S1-1 and Table S1-2).

Surprisingly, the organ coefficients of ovary, spleen, and kidney from control and D-gal+PC groups were higher than those of the D-gal group (*P* < 0.05, Figure 1B and Table S1-2). These data indicate that D-gal may impair the ovary, spleen, and kidney, and that this damage can be reversed partially by PC. We next examined the age-reversing effect of PC in the reproductive system of D-gal-induced aging mice.

### PC rescued oocyte morphology and developmental competence in D-gal-induced aging mice

**Figure 2:**
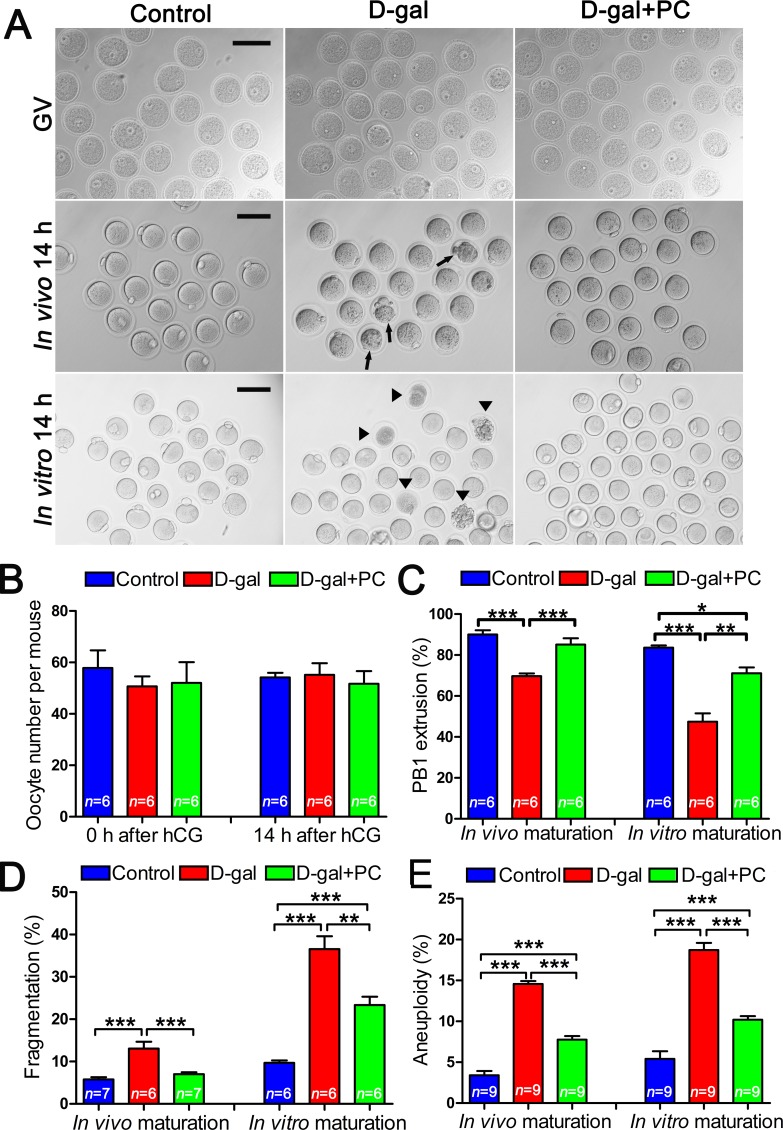
Impaired oocyte quality and developmental competence in D-gal-induced aging mice could be rescued by PC **A.** Morphology of oocytes at the GV stage, 14 hours after *in vivo* maturation and 14 hours after *in vitro* maturation. Arrows and triangles indicate morphologically abnormal oocytes after *in vivo* and *in vitro* maturation, respectively. Scale bar = 100 μm. **B.** There were no significant differences in terms of oocyte numbers per mouse before or 14 hours after hCG injection in the control, D-gal, and D-gal+PC groups. **C.** The percent of oocyte polar body extrusion was decreased in D-gal-induced aging mice. This inhibition was reversed by PC. **D.** D-gal-induced aging increased the percentage of oocyte fragmentation. This was decreased by PC administration after *in vivo* or *in vitro* maturation. **E.** D-gal-induced aging induced oocyte aneuploidy, which was decreased by PC after *in vivo* or *in vitro* maturation. Data are presented as the means ± SEMs and were processed by one-way ANOVA and Newman-Keuls post hoc tests. Significant differences between groups, *P < 0.05; **P < 0.01; ***P < 0.001. *n* indicates the number of mice for each treatment.

We assessed oocytes at the germinal vesicle (GV) and metaphase II (MII) stages generated from *in vivo* or *in vitro* maturation by examining their morphology with bright-field microscopy. We found that increased percentages of abnormal oocytes from the D-gal group matured both *in vivo* and *in vitro*. These oocytes were characterized by enlarged perivitelline spaces, fragmented or dark cytoplasm, or giant polar bodies, all of which are considered morphological abnormalities [33]. Conversely, after PC administration most D-gal-induced oocytes showed normal morphology, similar to the control group (Figure 2A).

In superovulated mice, the number of GV-stage oocytes retrieved from ovaries in the control, D-gal, and D-gal+PC groups were not statistically different. Similarly, the numbers of MII -stage oocytes collected from oviduct ampullae were comparable among the three groups (Figure 2B and Table S2-1).

In terms of first polar body (PB1) extrusion from *in vivo* and *in vitro* matured oocytes, we observed significant differences. During *in vivo* maturation, D-gal severely decreased PB1 extrusion, resulting in a much lower frequency compared to that of the control (*P* < 0.001). Interestingly, PC could apparently reverse impaired PB1 extrusion to rates comparable to the control group (control *vs* D-gal+PC, *P* > 0.05; D-gal *vs* D-gal+PC, *P* < 0.001). Similarly, the percentage of PB1 extrusion from oocytes matured *in vitro* was decreased by D-gal compared to control (*P* < 0.001), and administration of PC improved PB1 extrusion (D-gal *vs* D-gal+PC, *P* < 0.01). However, treatment did not rescue to the level of the control (control *vs* D-gal+PC, *P* < 0.05) (Figure 2C and Table S2-2).

We also calculated the percentages of fragmented oocytes matured *in vivo*. We calculated that 13.08% of fragmented oocytes matured in the D-gal group, which was much higher than the control group (*P* < 0.001). The rate of fragmentation was decreased in the D-gal+PC group to the control group (control *vs* D-gal+PC, *P* > 0.05; D-gal *vs* D-gal+PC, *P* < 0.001). For oocytes matured *in vitro*, 36.57% were fragmented in the D-gal group, which was much higher than that of the control group (*P* < 0.001). This impairment was partially reversed by PC (*P* < 0.01), but not to the level of control (*P* < 0.001) (Figure 2D and Table S2-3).

As age-related infertility is associated with chromosome aneuploidy [34], we checked if D-gal-induced aging could increase oocyte aneuploidy. For both the *in vivo* and *in vitro* maturation models, we found that the aneuploidy rate of sister chromatids was higher in the D-gal group compared to control (*P* < 0.001). After PC administration, decreased percentages of aneuploidy oocytes were observed both *in vivo* and *in vitro* (*P* < 0.001). However, PC administration could not normalize aneuploidy to the level of the control group (*P* < 0.001) (Figure 2E and Table S2-4). These results indicate that PC could reverse the deterioration of oocyte maturation in D-gal-induced aging mice.

### PC rescued spindle-chromosome complex (SCC) malformation in D-gal-induced aging mice

**Figure 3:**
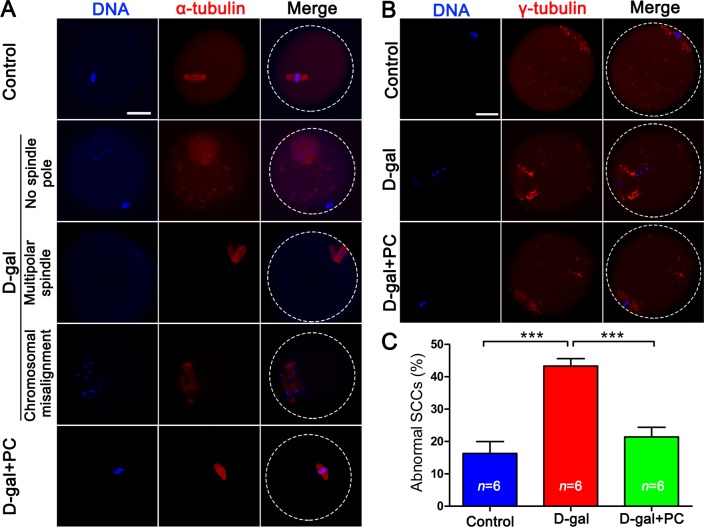
PC rescued SCC malformation and cytoskeletal abnormalities in D-gal-induced aging mice **A.** D-gal could induce various abnormal SCCs including no or multipolar spindle poles and chromosome misalignment in MII oocytes. These abnormalities were reversed by PC. Blue, DNA; red, α-tubulin; scale bar = 20 μm. **B.** Abnormal distribution of γ-tubulin in MII oocytes in D-gal-induced mice could be reversed by the administration of PC. Blue, DNA; red, γ-tubulin; scale bar = 20 μm. **C.** Malformed oocyte SCCs in D-gal-induced aging mice were rescued by PC. Data are presented as the means ± SEMs and were processed by one-way ANOVA and Newman-Keuls post hoc tests. Significant differences between groups, ***P < 0.001. *n* indicates the number of mice for each treatment.

PB1 extrusion failure and oocyte fragmentation in D-gal-induced aging mice may be associated with disordered spindle assembly and chromosome alignment. We observed defective spindles in the D-gal group. α-Tubulin labeling revealed that some oocytes had no spindle pole, while others had multiple spindle poles. Chromosome alignment in the D-gal group was also defective. The mid-plate, which appeared in control MII oocytes, was absent in the D-gal group, replaced by dispersed chromosomes in the mid-plate area. Notably, PC reversed all these malfunctions (Figure 3A).

We also examined SCC integrity by analyzing the distribution of γ-tubulin, which should be localized at the spindle pole area. In D-gal-treated mice, most oocytes lacked specific γ-tubulin localization at the spindle pole, but this was normalized by PC (Figure 3B).

We compared the frequency of abnormal SCC formation among the three groups. Almost half (43.32%) of oocytes generated from D-gal-treated mice had abnormal SCC (*P* < 0.001). This was halved by PC administration (*P* < 0.001), which was comparable to the control group (Figure 3C and Table S3).

Aging is associated with oocyte fragmentation and impaired PB1 extrusion. Abnormal SCC formation and translocation is the main reason for oocyte maturation failure and aneuploidy. These data suggest that PC may improve oocyte quality by correcting SCC formation and translocation, leading to the production of high-quality mature oocyte with a lower rate of aneuploidy.

### D-gal and PC do not influence telomere length or telomerase activity

**Figure 4:**
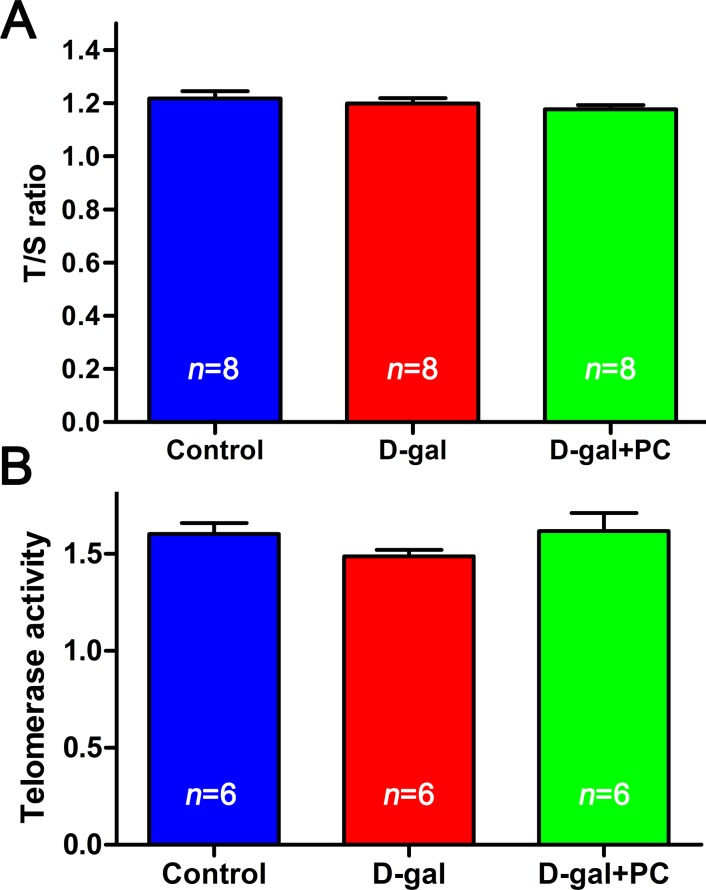
D-gal and PC did not affect telomere length or telomerase activity in mouse ovaries **A.** Relative telomere length shown as the T/S ratio determined by qPCR analysis. Administration of D-gal or PC had no effect. **B.** Telomerase activity of ovaries assessed with by ELISA. Administration of D-gal or PC had no effect. Data are presented as the means ± SEMs and were processed by one-way ANOVA and Newman-Keuls post hoc tests. *n* indicates the number of mice for each treatment.

Since short telomeres are considered a biomarker of chronic oxidative stress and biological aging [35, 36], we measured telomere length and telomerase activity prior to and after D-gal and PC treatment. Telomere length (indicated by T/S ratio) were not significantly different among the three groups (Figure 4A and Table S4). Similarly, no significant difference was observed in terms of telomerase activity in the whole ovary (Figure 4B and Table S4). Telomere length is considered a useful biomarker in determining biological and chronological aging [37, 38]. Our results showed that neither telomere length nor the telomerase activity was altered, indicating that telomeres were not disturbed in D-gal-induced aging mice.

### PC rescued antioxidant gene expression and antioxidant enzyme activity

**Figure 5:**
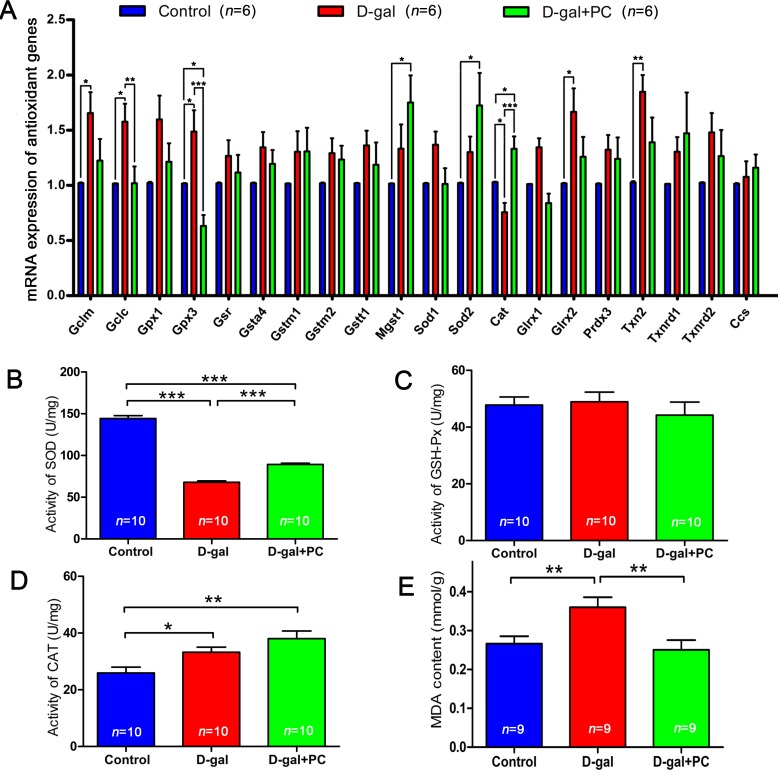
PC rescued antioxidant genes expression and enzymes activity **A.** Relative expression levels of antioxidant genes in ovaries by qPCR. D-gal significantly increased *Gclc* and *Gpx3* expression and decreased *Cat* expression, and PC reversed these alterations. **B.** SOD activity in ovaries was decreased by D-gal and partially increased after PC administration. **C.** GSH-Px activity in ovaries was not changed by D-gal or PC administration. **D.** CAT activity in ovaries was increased by D-gal and PC administration. **E.** MDA content in ovaries increased after D-gal treatment and decreased after PC administration. Data are presented as the means ± SEMs and were processed by one-way ANOVA and Newman-Keuls post hoc tests. Significant differences between groups, *P < 0.05; **P < 0.01; ***P < 0.001. *n* indicates the number of mice for each treatment.

As aging is highly correlated with the expression and activity of antioxidant genes and enzymes, we used quantitative real-time reverse transcription polymerase chain reaction (RT-PCR) to measure the ovarian mRNA levels of the antioxidant genes *Gclm*, *Gclc*, *Gpx1*, *Gpx3*, *Gsr*, *Gsta4*, *Gstm1*, *Gstm2*, *Gstt1*, *Mgst1*, *Sod1*, *Sod2*, *Cat*, *Glrx1*, *Glrx2*, *Prdx3*, *Txn2*, *Txnrd1*, *Txnrd2*, and copper chaperone for SOD (*Ccs*). D-gal significantly changed expression of some genes such as *Gclc*, *Gpx3,* and *Cat,* and PC reversed these alterations. However, D-gal changed the expression of some other genes such as *Gclm*, *Glrx2,* and *Txn2*, but these changes were not normalized by PC. Expression levels of most of the genes (*Gpx1*, *Gsr*, *Gsta4*, *Gstm1*, *Gstm2*, *Gstt1*, *Sod1*, *Glrx1*, *Prdx3*, *Txnrd1*, *Txnrd2,* and *Ccs*) were not affected by D-gal or PC treatments. Interestingly, the expression levels of *Mgst1* and *Sod2* were not changed after D-gal treatment but increased after PC administration (Figure 5A and Table S5-1).

We also measured antioxidant enzyme levels and activities. Superoxide dismutase (SOD) content was decreased after D-gal treatment (*P* < 0.001), and PC partially reversed this decrease (control *vs* D-gal, *P* < 0.001; control *vs* D-gal+PC, *P* < 0.001) (Figure 5B and Table S5-2). There was no significant difference in glutathione peroxidase (GSH-Px) activity among the three groups (Figure 5C and Table S5-3). Catalase (CAT) content was higher in the D-gal group than in the control group (*P* < 0.05), and this increase could not be reversed by PC administration (control *vs* D-gal+PC, *P* < 0.01) (Figure 5D and Table S5-4). Methane dicarboxylic aldehyde (MDA) content was also measured as a biomarker for oxidative stress and aging. MDA was higher in the D-gal group compared to control (*P* < 0.01), and this increase was inhibited by PC (*P* < 0.01) (Figure 5E and Table S5-5).

Consistent with the generally accepted view that antioxidant gene expression and antioxidant enzyme activity are involved in the aging process [39, 40], we found alterations in some genes and enzymes. Moreover, MDA was increased in the D-gal group and could be inhibited by PC. This led us to explore whether mitochondria and ROS are downstream of PC's effects.

### Aggregated mitochondrial distribution in D-gal-induced oocytes was normalized by PC

**Figure 6:**
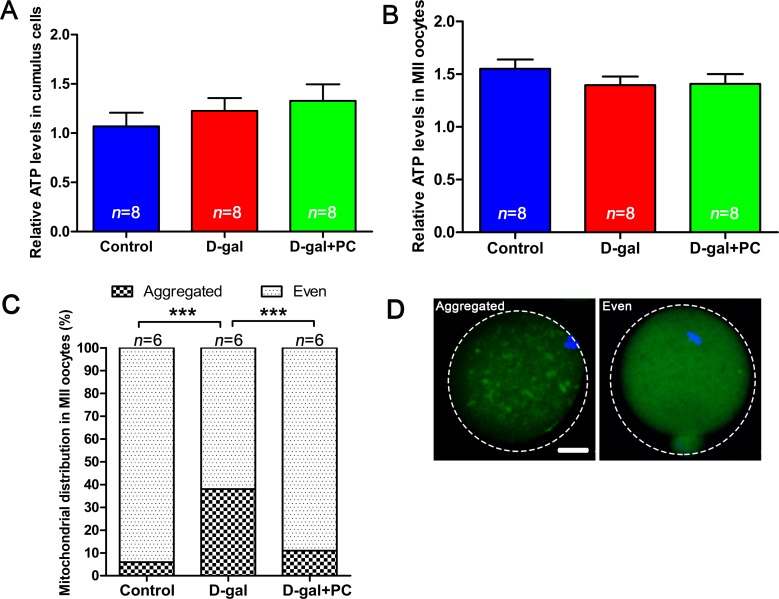
Mitochondrial distribution but not ATP level was altered by D-gal and reversed by PC **A.** Relative ATP levels in cumulus cells and **B.** relative ATP levels in MII oocytes were not affected by D-gal or PC administration. Data are presented as means ± SEMs. **C.** Percentages of MII oocytes with altered mitochondrial distribution after D-gal injection and PC administration. Column with large square, aggregated mitochondria distribution; column with small square, even mitochondria distribution. Data are presented as means. **D.** Representative images of mitochondria with aggregated or even distribution. Data were processed by one-way ANOVA and Newman-Keuls post hoc tests. Significant differences between groups, ***P < 0.001. Scale bar = 20 μm. *n* indicates the number of mice for each treatment.

Next, we examined ATP levels and mitochondrial distribution in MII oocytes. Relative ATP levels in cumulus cells were not significantly different in the D-gal or D-gal+PC groups compared to control (Figure 6A and Table S6-1). A similar result was observed in oocytes (Figure 6B and Table S6-1). However, we observed a significant difference when we assessed mitochondrial distribution in MII oocytes. The distribution of mitochondria was classified as “aggregated” or “even.” Only 5.76% of MII oocytes exhibited aggregated distribution in the control group. However, after D-gal administration, this proportion increased to 37.97%, which was significantly higher than control (*P* < 0.001). After PC administration, 88.95% of oocytes were evenly distributed around mitochondria, which was similar to the oocytes in control (Figure 6C and 6D, Table S6-2). Mitochondrial distribution is a cytoskeleton-dependent intracellular traffic behavior [41]. The distribution of mitochondria in oocytes determines spindle translocation [42]. Although ATP levels in the whole oocytes were not changed, abnormal distribution of mitochondria induced by D-gal and later recovered by PC may be associated with oocyte maturation. Furthermore, ROS accumulation can damage the cytoskeleton and affect mitochondrial distribution [13, 19], which led us to evaluate the ROS level in D-gal- or PC-treated oocytes.

### PC reduced high ROS levels induced by D-gal in MII oocytes

**Figure 7:**
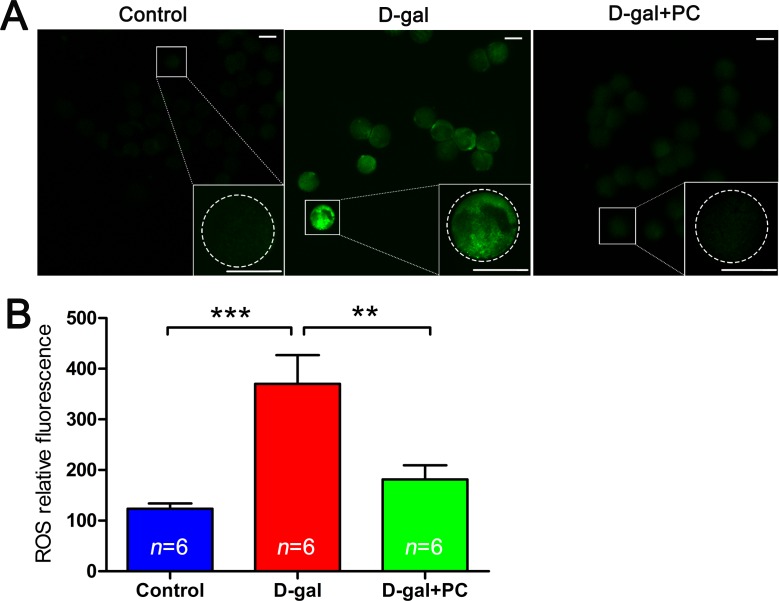
PC inhibited ROS level in MII oocytes in D-gal-induced aging mice **A.** Representative images of ROS generation determined by DCFH-DA fluorescence (green). Scale bar = 50 μm. **B.** ROS relative fluorescence intensity. ROS generation in oocytes was obviously increased after D-gal treatment; this was suppressed to the control group level after PC administration. Data are presented as the means ± SEMs and were processed by one-way ANOVA and Newman-Keuls post hoc tests. Significant differences between groups, **P < 0.01; ***P < 0.001. *n* indicates the number of mice for each treatment.

ROS levels can indicate oxidative stress. In oocytes, we measured intracellular ROS levels in *in vivo* matured MII oocytes. Dichlorofluorescein diacetate (DCFH-DA) fluorescence intensity was significantly higher in the MII oocytes of the D-gal-treated group than in the control or D-gal+PC groups, indicating enhanced ROS production after D-gal administration (Figure 7A). We quantified the relative fluorescence intensities and confirmed that ROS levels were much higher in the D-gal group (*P* < 0.001). Interestingly, when PC was administered, ROS levels decreased to a value comparable to the control group (Figure 7B and Table S7). These results suggest that D-gal significantly increases ROS generation in oocytes. Significantly, PC reduced ROS levels in the MII oocytes of D-gal-induced aging mice (*P* < 0.01).

### PC inhibited D-gal-induced early apoptosis in MII oocytes

**Figure 8:**
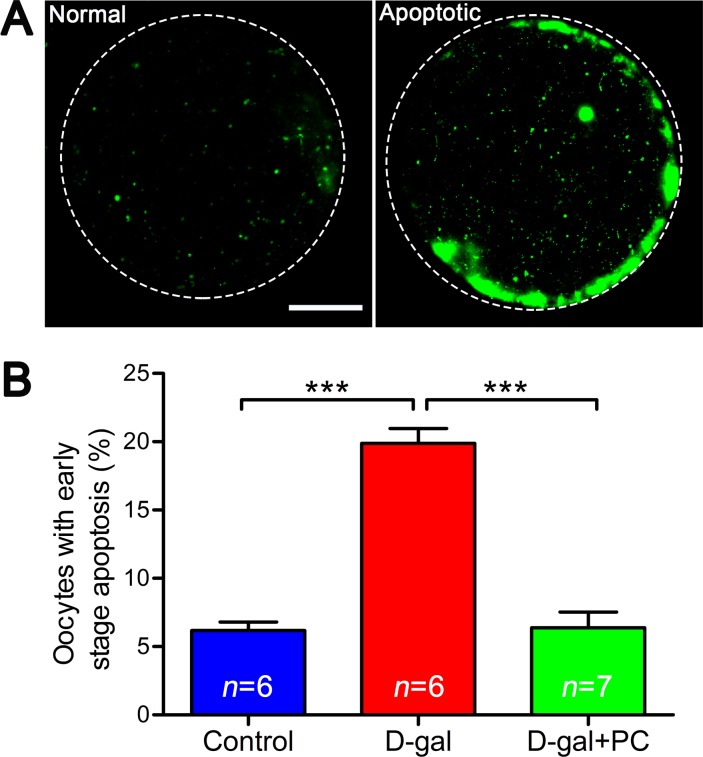
PC inhibited early stage apoptosis in MII oocytes in D-gal-induced aging mice **A.** Representative images of early stage apoptosis in MII oocytes. Oocytes without green fluorescence signals at the zona pellucida and oocyte membrane were non-apoptotic, and oocytes undergoing early apoptosis were characterized by a clear green signal in the zona pellucida and membrane. Scale bar = 20 μm. **B.** Percent oocytes undergoing early stage apoptosis. D-gal induced early stage apoptosis in oocytes, and this was inhibited by PC. Data are presented as the means ± SEMs and were processed by one-way ANOVA and Newman-Keuls post hoc tests. Significant differences between groups, ***P < 0.001. *n* indicates the number of mice for each treatment.

High levels of ROS can induce apoptosis. We therefore performed annexin-V staining to determine whether the frequency of early stage apoptosis in oocytes was altered by D-gal and PC treatment. Oocytes undergoing early apoptosis were characterized by a clear green signal in the membrane and zona pellucida (Figure 8A). We quantified the fluorescence signals and found that 19.89% of oocytes in the D-gal group were apoptotic (*P* < 0.001). After PC administration, the percentage of apoptotic cells decreased (control *vs* D-gal+PC, *P* > 0.05; D-gal *vs* D-gal+PC, *P* < 0.001) (Figure 8B and Table S8). Oocyte apoptosis is always accompanied by abnormal morphology changes, which was verified by our previous results. These indicate that D-gal triggers apoptosis in MII oocytes, which is inhibited by PC.

### PC rescued litter size in D-gal-treated mice

**Figure 9:**
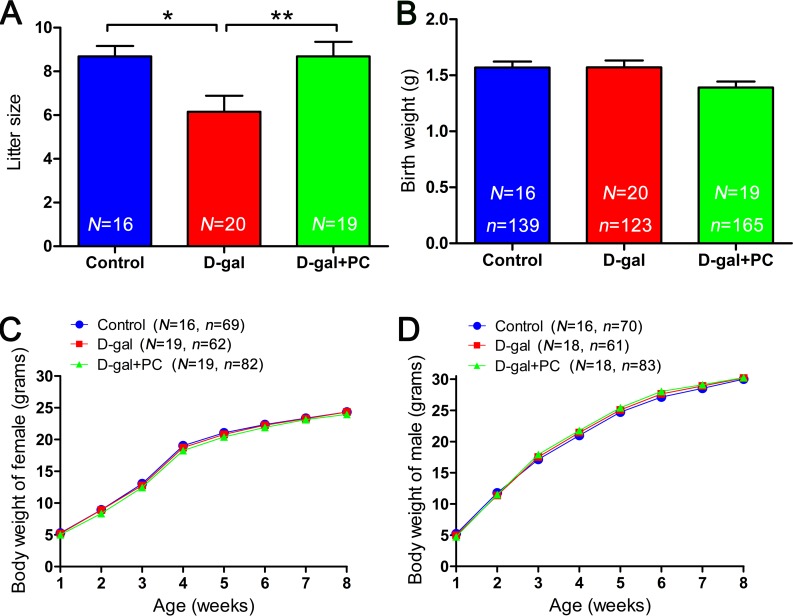
PC treatment led to increase in the litter size of D-gal-induced aging mice but did not influence offspring birth weight or growth rate **A.** Litter sizes in the control, D-gal, and D-gal+PC groups. **B.** Birth weights in the control, D-gal and D-gal+PC groups. **C.** Postnatal growth of female from weeks 1 to 8 in the control, D-gal, and D-gal+PC groups. **D.** Postnatal growth of male from weeks 1 to 8 in the control, D-gal, and D-gal+PC groups. Data are presented as the means ± SEMs for **A.** and **B.** and means for **C.** and **D.**. Data were processed by one-way ANOVA and Newman-Keuls post hoc tests. Significant differences between groups, *P < 0.05; **P < 0.01. *N* indicates the number of female mice with plugs and that gave birth for each treatment; *n* shows the total number of offspring for each treatment.

Finally, the numbers of offspring in the three groups were evaluated. Each normal female in the control group delivered an average of 8.69 pups. The D-gal-induced aging female exhibited low reproductive ability compared with the control group (*P* < 0.05). Significantly, PC treatment increased the litter size in D-gal-induced aging mice to the level of the controls (control *vs* D-gal+PC, *P* > 0.05; D-gal *vs* D-gal+PC, *P* < 0.01) (Figure 9A and Table S9-1). Offspring birth weight was not significantly different among the three groups (Figure 9B and Table S9-2). Considering that aging mice have a high proportion of birth defects correlated with aneuploidy, we assessed the growth state of offspring in both female (Figure 9C and Table S9-3) and male (Figure 9D and Table S9-4) and detected no significant differences among the three groups. These results indicate that PC has no detectable side effects on the offspring of D-gal-induced aging mice.

## DISCUSSION

In this study, we investigated the ability of PC to reverse D-gal-induced reproduction impairments in mice. Our results indicate that consecutive subcutaneous D-gal injections had toxic effects on the female reproductive system. Notably, PC protects against the D-gal-induced reduction of fertility in mice.

D-gal-induced aging is associated with oxidative stress and AGE generation [43]. ROS play key roles in this process as their accumulation leads to the induction and maintenance of cellular senescence [44]. In humans, aging is associated with increased ROS levels and decreased antioxidant levels in oocytes, cumulus cells, and follicular fluid [45-47], which impair multiple physiological processes from oocyte maturation to fertilization, embryo development, and pregnancy [48].

We observed that D-gal treatment reduced ovary and oocyte quality. These data concur with previous reports that D-gal can damage ovarian function in mice [49] and that oocyte quality decreases with age [50]. Failure of polar body extrusion is often correlated with abnormal spindle formation and chromosome congression. We observed disrupted oocyte SCC integrity and an increased percentage of aneuploidy after D-gal treatment. This is probably a consequence of increased ROS levels. In natural ovulation, aging decrease oocyte number by altering hormone levels [51]. Unexpectedly, D-gal or PC administration did not influence oocyte number after gonadotropin stimulation. We propose that in gonadotropin-treated mice, age-related immature follicles will respond to this stimulation, inducing more oocyte to ovulate [52]. Interestingly, ROS levels were decreased after PC treatment, and this was accompanied by improved ovarian function and oocyte quality. Therefore, PC appears to protect against D-gal-induced ovarian aging by reducing ROS levels.

Telomere dysfunction may contribute to reproductive aging-associated meiotic defects, miscarriage, and infertility [53-55]. Unexpectedly, we did not observe any D-gal- or PC-induced changes in telomerase activity or the T/S ratio in ovaries. We propose that telomere dysfunction is mainly correlated with natural aging and might be observed over a longer term. There may not have been enough time for telomeres to shorten after only 40 days of D-gal treatment in our protocol.

Mammalian ovaries possess antioxidant defenses including the antioxidant tripeptide glutathione (GSH), and ROS-scavenging enzymes such as SOD, GSH-Px, CAT, glutathione S-transferase (GST), and peroxiredoxin (PRDX) [4, 56, 57]. We hypothesized that ovarian aging is associated with decreased expression of ovarian antioxidant genes and lower antioxidant enzyme levels, resulting in oxidative damage to ovarian function. This was confirmed by the observed variation of SOD activity and oxidative stress marker MDA content after D-gal treatment. Consistently, D-gal increased the expressions of *Gclc* and *Gpx3* and decreased that of *Cat*. PC was able to reverse these changes, indicating that its likely rescue mechanism is related to the inhibition of ROS production.

To date, there is no evidence that PC directly affects mitochondrial distribution. We speculate that PC improved mitochondrial distribution mainly *via* its inhibition of ROS. A previous study reported that mitochondrial distribution is a cytoskeleton-dependent intracellular traffic behavior that relies on microtubule organization [41]. The disruption of mitochondrial distribution is therefore a consequence of malfunctioned cytoskeleton. ROS deteriorate microtubule dynamics and lead to their instability [58], which further deranges mitochondrial distribution and function. Galactitol is a secondary metabolite of D-gal formed by the reduction of D-gal after cellular metabolism. Gradual intracellular accumulation of galactitol can increase osmotic stress and elevate ROS levels [59]. Binding between PC and ROS can form non-radical products, thus preventing ROS-mediated damage of organelles including microtubules [60]. Therefore, PC treatment can prevent ROS from decreasing microtubule stability and help maintain proper mitochondrial distribution.

ROS serve as both key signaling molecules in physiological processes such as meiotic resumption and as indicators of cell apoptosis [48]. Apoptotic pathways are also activated by ROS production [20]. Our results indicate that D-gal treatment in mice triggers early stage apoptosis in MII oocytes, possibly due to ROS accumulation. Any treatment that inhibits ROS production, including PC, may decrease apoptosis.

Although no significant differences were found in birth weight, the results of our oocyte and ovary analyses suggest that PC can rescue D-gal-induced impairment of reproduction, especially small litter size. In conclusion, our results show that PC can help maintain reproductive performance in a D-gal-induced aging model. It is important to note that data obtained from a mouse model may not extrapolate directly to human reproduction and natural aging, and more extensive research is needed in a natural aging model before any clinic trials are to be attempted.

**Figure 10:**
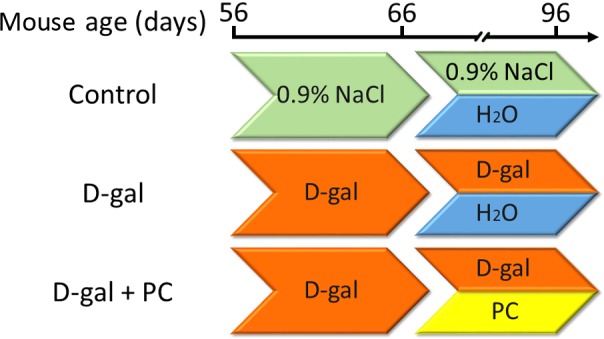
Experimental design B6D2F/1 mice at 8 weeks of age were used. (1) The control group was subcutaneously injected with 0.9% saline (green) and intragastrically administered ultrapure water daily (blue). (2) The D-gal group was subcutaneously injected with 250 mg/kg/day D-gal solution (orange) and intragastrically administered ultrapure water daily. (3) The D-gal+PC group was subcutaneously injected with 250 mg/kg/day D-gal solution and intragastrically administered 500mg/kg/day of PC daily (yellow).

## MATERIALS AND METHODS

### Ethics statement

All experiments adhered with the National Research Council Guide for the Care and Use of Laboratory Animals and were approved by the Institutional Animal Care and Use Committee at Inner Mongolia University.

### Experimental design, mouse feeding, mating, and offspring assessment

Female B6D2F1 mice were purchased from the Research Center for Laboratory Animal Science of Inner Mongolia University and housed under 12:12-h light:dark cycles in a specific pathogen-free animal facility at the Research Center for Laboratory Animal Science of Inner Mongolia University. During the whole experiment, mice had free access to food and water. At the age of 8 weeks, adult female mice were randomly divided into three groups and treated as follows. In the control group, mice were subcutaneously injected daily with 0.1 mL 0.9% saline for up to 40 days. During the last 30 days, 0.4 mL ultrapure water was intragastrically administered each day. For the D-gal group, mice were subcutaneously injected with 250 mg/kg/day D-gal (Sigma Aldrich, St. Louis, MO, USA) solution for up to 40 days. During the last 30 days, 0.4 mL ultrapure water was administered intragastrically each day. For the D-gal+PC group, mice were subcutaneously injected with 250 mg/kg/day D-gal solution for up to 40 days. During the last 30 days, 500 mg/kg/day PC (Binmei Biotechnology, Zhoushan, China) was intragastrically administered each day. A schematic of the experimental design is shown in Figure 10. Unless otherwise stated, all chemicals and media were purchased from Sigma Aldrich.

Male mice at 12 weeks of age with proven fertility were used for mating. Late in the afternoon, a single female mouse was placed in the cage of one male for mating. On the following morning, successfully mated female mice with a plug were returned to their cages for pup delivery. Female without mating plugs were not used for breeding experiments. The number and weight of offspring were recorded immediately after delivery. Body weight was measured and recorded once a week through the eighth week.

### Ovary, liver, spleen, and kidney weight and oocyte collection

GV-stage oocytes were collected by puncturing the follicles of ovaries 48 hours after injection of pregnant mare serum gonadotropin (PMSG, SanSheng, Ningbo, China). Cumulus cells were removed by gentle pipetting. Oocytes were washed thoroughly and cultured in Chatot-Ziomet-Bavister (CZB) under liquid paraffin oil at 37°C in an atmosphere of 5% CO_2_ in air for 14 hours until they reached the MII stage.

For *in vivo* MII-stage oocyte collection, mice were superovulated with 10 IU PMSG followed 48 h later by 10 IU human chorionic gonadotropin (hCG, SanSheng). MII oocytes with cumulus mass were released from the oviduct ampullae 14 h after hCG injection. Cumulus cells were dispersed by 0.3 mg/mL hyaluronidase in HEPES-M2 medium. Oocytes were cultured in CZB medium for 30 min of recovery. All oocytes from *in vivo* or *in vitro* maturation were examined for PB1 extrusion and fragmentation. The criteria for fragmentation evaluation were based on previous reports [61, 62]. Body weight and organ (ovary, liver, and spleen) wet weight were measured and recorded at the end of treatment, and ovaries were kept frozen at −80°C for further experiments.

### Immunofluorescence microscopy and chromosome spreading

Oocytes were exposed to acidic tyrode solution (pH 2.5) for a few seconds to remove the zona pellucida followed by three washes in M2 medium. Oocytes were then fixed in 4% paraformaldehyde (Electron Microscopy Sciences, Hatfield, PA, USA) in phosphate-buffered saline (PBS) at room temperature for 30 min, followed by permeabilization in PBS containing 0.5% Triton X-100 for 2 h at room temperature. Sample blocking was conducted with 1% bovine serum albumin (BSA, Amresco, Solon, OH, USA) in PBS containing 1/1000 Tween-20 (Amresco) and 1/10,000 Triton X-100. After blocking, samples were incubated with primary antibodies overnight at 4°C. For primary antibodies, we used mouse anti-alpha tubulin (1:2000, Abcam, Cambridge, UK) and anti-gamma tubulin (1:500, Abcam). For secondary antibodies, we used DyLight 549-conjugated donkey anti-mouse (1:100, Jackson ImmunoResearch Laboratories, West Grove, PA, USA). DNA was stained with 4′,6-diamidino-2-phenylindole (DAPI, 5 μg/mL, Roche, Mannheim, Germany) for 10 min. After staining and washing, samples were mounted on glass slides using Vectashield mounting medium (Vector Labs, Burlingame, CA, USA) and examined with a confocal laser-scanning microscope (Nikon, A1R, Tokyo, Japan). Images were analyzed with NIS-Element AR 3.0 software. Chromosome spreading analysis was performed as we described previously [63].

### Telomere length measurement and telomerase activity assay

The average telomere length was measured from total genomic DNA of ovaries using a real-time PCR assay. The reactions were performed with telomeric primers for a reference control gene (the mouse 36B4 single-copy gene) using PCR settings as previously described [64]. For each PCR reaction, a standard curve was made by serial dilutions of known amounts of DNA from the same tissues. The telomere signal was normalized to the signal from the single-copy gene to generate a T/S ratio indicative of relative telomere length.

The telomerase activity of ovaries was measured with a telomerase enzyme-linked immunosorbent assay (ELISA) kit (Cusabio, Wuhan, China). The experiments were performed according to the manufacturer's instructions. In brief, a telomerase-specific antibody was pre-coated onto a microplate. Standards and samples were pipetted into the wells, and any telomerase present was bound by the immobilized antibody. After removing any unbound substances, a biotin-conjugated antibody specific for telomerase was added to the wells. After washing, avidin-conjugated horseradish peroxidase was added to the wells. Following a wash to remove any unbound avidin-enzyme reagent, a substrate solution was added, and color developed in proportion to the amount of telomerase bound in the initial step. After color development was terminated, color intensity was measured with a high-sensitivity luminometer (Thermo Scientific, Waltham, MA, USA).

### Quantitative real-time RT-PCR and enzyme assay

For quantitative real-time RT-PCR, total RNA was extracted from ovaries using the TaKaRa MiniBEST Universal RNA Extraction Kit (TaKaRa, Dalian, China) according to the manufacturer's instructions. cDNA was synthesized using the PrimeScript RT reagent Kit (TaKaRa) following the manufacturer's instructions. The primer sequences were obtained from a previous publication [4]. RT-PCR was performed with the SYBR Green kit (TaKaRa). The comparative Ct method was used for data analysis, and *Gapdh* was used as an internal control.

For ovarian enzyme activity assays and MDA measurement, all procedures were performed according to the manufacturer's instructions with kits purchased from the Nanjing Jiancheng Bioengineering Institute (Nanjing, China) unless noted otherwise.

### ATP measurement

ATP levels in oocytes and cumulus cells were measured using a kit (FL-ASC from Sigma Aldrich) as previously described with a minor change [65]. Briefly, 30 denuded oocytes or cumulus cells collected from 30 cumulus-oocyte complexes in each mouse were snap-frozen in a microfuge tube containing 50 μL water and stored at −80°C. For ATP assays, 50 μL of each thawed sample solution was added to 100 μL ice-cold Cell ATP-Releasing Reagent and incubated on ice for 5 min, followed by the addition of 100 μL ice-cold ATP Assay Mix (1:25 diluted in assay mix buffer). The reaction mixture was then incubated for 10 min in the dark at room temperature for the initial chemiluminescence flash period. The bioluminescence of each sample was measured with a high-sensitivity luminometer (Thermo Scientific).

### Evaluation of mitochondrial distribution

Denuded oocytes were fixed in 4% paraformaldehyde in PBS for 30 min in a humidified chamber, washed, and incubated in 25 nM Mitotracker Green-Fluorescence Mitochondria (Mitotracker Green FM; Molecular Probes, Eugene, OR, USA) in PBS-BSA for 30 min in the dark. After several washes, the oocytes were transferred into a drop of medium containing DAPI for 10 min. After staining, the samples were mounted on glass slides using Vectashield (Vector Labs) mounting medium and examined with a confocal laser-scanning microscope (Nikon). Images were analyzed with NIS-Element AR3.0 software.

### Determination of ROS generation

To assess ROS production, cumulus-denuded oocytes were incubated with an oxidation-sensitive fluorescent probe (dichlorofluorescein, DCFH) for 30 min at 37°C in CZB containing 10 μM DCFH-DA (Nanjing Jiancheng Bioengineering Institute). Then the oocytes were washed three times with D-PBS with 0.1% BSA and mounted on glass slides. Florescence intensity in each oocyte was measured with a confocal system (Nikon) with the same scan settings for each sample.

### Apoptosis detection

Apoptosis detection was performed with an annexin-V staining kit (Vazyme, Nanjing, China) according to the manufacturer's instructions. Briefly, oocytes were washed twice in PBS and stained for 10 min in the dark with 100 mL binding buffer that contained 10 mL annexin-V-FITC. The samples were observed immediately after staining. Fluorescent signals were measured using a fluorescent microscope (Nikon) with 450-490 nm (excitation) and 520 nm (emission) filters.

### Statistical analysis

At least six replicates were conducted for each treatment. Results are shown as means ± SEMs. Statistical comparisons were made using analysis of variance (ANOVA), and differences between treatment groups were assessed with Newman-Keul's multiple comparison post hoc tests. All analyses were performed using GraphPad Prism 5.0 statistical software (GraphPad Software Inc., La Jolla, CA, USA). *P* < 0.05 was considered significant.

## SUPPLEMENTARY MATERIAL TABLES


