# Supplementary material for: C-phycocyanin protects against low fertility by inhibiting reactive oxygen species in aging mice
Source: Oncotarget. 2016 Mar 17;7(14):17393–409. doi: 10.18632/oncotarget.8165 (PMC4951220; doi:10.18632/oncotarget.8165)
Supplement: Supplementary file 1 [file oncotarget-07-17393-s001.pdf]

C-phycocyanin protects against low fertility by inhibiting reactive oxygen species in aging mice

Supplementary Material

Table S1-1. PC did not affect organ weight in D-gal-induced aging mice

Treatment	Organ weight (mg) Mean ± SEM (*n*)			
	Ovary	Liver	Spleen	Kidney
Ctrl	44.95 ± 1.11 (6)[a]	1535.92 ± 20.36 (6)[a]	104.33 ± 3.37 (6)[a]	347.73 ± 9.09 (6)[a]
D-gal	40.40 ± 1.17 (6)[a]	1506.22 ± 94.31 (6)[a]	99.47 ± 4.26 (6)[a]	353.48 ± 12.43 (6)[a]
D-gal+PC	41.77 ± 3.16 (6)[a]	1427.40 ± 76.66 (6)[a]	95.23 ± 4.94 (6)[a]	323.30 ± 12.73 (6)[a]

Within column, data without common superscript letters indicate significant difference ($P < 0.05$). *n* indicates the number of mice for each treatment.

Table S1-2. PC reversed some organ coefficients in D-gal-induced aging mice

Treatment	Organ coefficient (mg/g) Mean ± SEM (*n*)			
	Ovary/body	Liver/body	Spleen/body	Kidney/body
Ctrl	1.54 ± 0.08 (6)[a]	52.31 ± 1.28 (6)[a]	3.55 ± 0.12 (6)[a]	11.82 ± 0.25 (6)[a]
D-gal	1.23 ± 0.05 (6)[b]	45.73 ± 3.28 (6)[a]	3.01 ± 0.14 (6)[b]	10.69 ± 0.35 (6)[b]
D-gal+PC	1.50 ± 0.11 (6)[a]	51.34 ± 2.26 (6)[a]	3.42 ± 0.13 (6)[a]	11.61 ± 0.15 (6)[a]

Within column, data without common superscript letters indicate significant difference ($P < 0.05$). *n* indicates the number of mice for each treatment.

Table S2-1. PC did not increase oocyte number in D-gal-induced aging mice

Treatment	Oocyte number per mouse Mean ± SEM (*n*)	
	0 h after hCG	14 h after hCG
Ctrl	57.83 ± 6.89 (6)[a]	54.17 ± 1.80 (6)[a]
D-gal	50.67 ± 3.92 (6)[a]	55.17 ± 4.56 (6)[a]
D-gal+PC	52.00 ± 8.12 (6)[a]	51.67 ± 4.96 (6)[a]

Within column, data without common superscript letters indicate significant difference ($P < 0.05$).
n indicates the number of mice for each treatment

Table S2-2. PC increased oocyte PB1 extrusion in D-gal-induced aging mice

Treatment	PB1 extrusion (%) Mean ± SEM (*n*)	
	in vivo maturation	*in vitro* maturation
Ctrl	90.03 ± 2.05 (6)[a]	83.61 ± 1.13 (6)[a]
D-gal	69.72 ± 1.29 (6)[b]	47.45 ± 4.13 (6)[b]
D-gal+PC	85.15 ± 3.09 (6)[a]	71.11 ± 2.82 (6)[c]

Within column, data without common superscript letters indicate significant difference (ab, $P < 0.001$; ac, $P < 0.05$; bc, $P < 0.01$).
n indicates the number of mice for each treatment.

Table S2-3. PC inhibited oocyte fragmentation in D-gal-induced aging mice

Treatment	Fragmentation (%) Mean ± SEM (*n*)	
	in vivo maturation	*in vitro* maturation
Ctrl	5.78 ± 0.52 (7)[a]	9.67 ± 0.58 (6)[a]
D-gal	13.08 ± 1.60 (6)[b]	36.57 ± 3.02 (6)[b]
D-gal+PC	7.01 ± 0.48 (7)[a]	23.34 ± 1.96 (6)[c]

Within column, data without common superscript letters indicate significant difference (ab, $P < 0.001$; ac, $P < 0.001$; bc, $P < 0.01$).
n indicates the number of mice for each treatment.

Table S2-4. PC inhibited oocyte aneuploidy in D-gal-induced aging mice

Treatment	Aneuploidy (%) Mean ± SEM (*n*)	
	in vivo maturation	*in vitro* maturation
Ctrl	3.41 ± 0.52 (9)[a]	5.41 ± 0.94 (9)[a]
D-gal	14.57 ± 0.35 (9)[b]	18.71 ± 0.88 (9)[b]
D-gal+PC	7.77 ± 0.43 (9)[c]	10.19 ± 0.44 (9)[c]

Within column, data without common superscript letters indicate significant difference ($P < 0.001$).
n indicates the number of mice for each treatment.

Table S3. PC normalized spindle chromosome complex in D-gal-induced aging mice

Treatment	Abnormal SCCs (%) Mean ± SEM (n)
Ctrl	16.31 ± 3.68 (6)[a]
D-gal	43.32 ± 2.28 (6)[b]
D-gal+PC	21.40 ± 3.01 (6)[a]

Data without common superscript letters indicate significant difference ($P < 0.001$).
n indicates the number of mice for each treatment.

Table S4. D-gal and PC did not influence ovary telomere length or telomerase activity

Treatment	T/S ratio Mean ± SEM (n)	Telomerase activity Mean ± SEM (n)
Ctrl	1.22 ± 0.03 (8)[a]	1.60 ± 0.06 (6)[a]
D-gal	1.20 ± 0.02 (8)[a]	1.49 ± 0.03 (6)[a]
D-gal+PC	1.18 ± 0.02 (8)[a]	1.62 ± 0.09 (6)[a]

Within column, data without common superscript letters indicate significant difference ($P < 0.05$).
n indicates the number of mice for each treatment.

Table S5-1. PC rescued the expression of some antioxidant genes

Gene name	Treatment (*n*) Mean ± SEM			superscript letters annotation
	Ctrl (6)	D-gal (6)	D-gal+PC (6)	
Gclm	1.02 ± 0.01^{a}	1.66 ± 0.19^{b}	1.22 ± 0.20^{ab}	ab, $P < 0.05$
Gclc	1.02 ± 0.00^{a}	1.58 ± 0.16^{b}	1.02 ± 0.15^{ac}	ab, $P < 0.05$; bc, $P < 0.01$
Gpx1	1.02 ± 0.01^{a}	1.60 ± 0.22^{a}	1.21 ± 0.17^{a}	$P > 0.05$
Gpx3	1.02 ± 0.00^{a}	1.49 ± 0.19^{b}	0.63 ± 0.10^{c}	ab, $P < 0.05$; ac, $P < 0.05$; bc, $P < 0.001$
Gsr	1.02 ± 0.01^{a}	1.27 ± 0.14^{a}	1.12 ± 0.16^{a}	$P > 0.05$
Gsta4	1.02 ± 0.01^{a}	1.35 ± 0.14^{a}	1.19 ± 0.13^{a}	$P > 0.05$
Gstm1	1.02 ± 0.00^{a}	1.30 ± 0.19^{a}	1.31 ± 0.22^{a}	$P > 0.05$
Gstm2	1.02 ± 0.01^{a}	1.29 ± 0.14^{a}	1.23 ± 0.13^{a}	$P > 0.05$
Gstt1	1.02 ± 0.00^{a}	1.36 ± 0.13^{a}	1.19 ± 0.20^{a}	$P > 0.05$
Mgst1	1.02 ± 0.00^{a}	1.33 ± 0.22^{ab}	1.75 ± 0.25^{b}	ab, $P < 0.05$
Sod1	1.02 ± 0.00^{a}	1.37 ± 0.12^{a}	1.01 ± 0.14^{a}	$P > 0.05$
Sod2	1.02 ± 0.00^{a}	1.30 ± 0.14^{ab}	1.73 ± 0.29^{b}	ab, $P < 0.05$
Cat	1.03 ± 0.01^{a}	0.76 ± 0.08^{b}	1.33 ± 0.11^{c}	ab, $P < 0.05$; ac, $P < 0.05$; bc, $P < 0.001$
Glrx1	1.01 ± 0.00^{a}	1.35 ± 0.08^{a}	0.84 ± 0.09^{a}	$P > 0.05$
Glrx2	1.02 ± 0.00^{a}	1.67 ± 0.21^{b}	1.26 ± 0.18^{ab}	ab, $P < 0.05$
Prdx3	1.02 ± 0.00^{a}	1.32 ± 0.13^{a}	1.24 ± 0.19^{a}	$P > 0.05$
Txn2	1.03 ± 0.01^{a}	1.85 ± 0.15^{b}	1.39 ± 0.22^{ab}	ab, $P < 0.01$
Txnrd1	1.01 ± 0.00^{a}	1.30 ± 0.13^{a}	1.47 ± 0.37^{a}	$P > 0.05$
Txnrd2	1.02 ± 0.01^{a}	1.48 ± 0.18^{a}	1.27 ± 0.24^{a}	$P > 0.05$
Ccs	1.01 ± 0.01^{a}	1.08 ± 0.14^{a}	1.16 ± 0.12^{a}	$P > 0.05$

Within row, data without common superscript letters indicate significant difference (see superscript letters annotation).

n indicates the number of mice for each treatment.

Table S5-2. PC increased SOD activity in D-gal-induced aging mice

Treatment	SOD activity (U/mg) Mean ± SEM (*n*)
Ctrl	144.30 ± 3.41 (10)^{a}
D-gal	67.92 ± 1.63 (10)^{b}
D-gal+PC	89.21 ± 1.49 (10)^{c}

Data without common superscript letters indicate significant difference ($P < 0.001$).

n indicates the number of mice for each treatment.

Table S5-3. D-gal or PC did not affect GSH-Px activity

Treatment	GSH-Px activity (U/mg) Mean ± SEM (*n*)
Ctrl	47.78 ± 2.84 (10)[a]
D-gal	48.92 ± 3.39 (10)[a]
D-gal+PC	44.23 ± 4.59 (10)[a]

Data without common superscript letters indicate significant difference ($P < 0.05$).
n indicates the number of mice for each treatment.

Table S5-4. PC did not reverse the increased CAT activity in D-gal-induced aging mice

Treatment	CAT activity (U/mg) Mean ± SEM (*n*)
Ctrl	25.92 ± 2.05 (10)[a]
D-gal	33.21 ± 1.83 (10)[b]
D-gal+PC	38.01 ± 2.74 (10)[bc]

Data without common superscript letters indicate significant difference (ab, $P < 0.05$; ac, $P < 0.01$).
n indicates the number of mice for each treatment.

Table S5-5. PC decreased MDA content in D-gal-induced aging mice

Treatment	MDA content (mmol/g) Mean ± SEM (*n*)
Ctrl	0.27 ± 0.02 (9)[a]
D-gal	0.36 ± 0.03 (9)[b]
D-gal+PC	0.25 ± 0.03 (9)[a]

Data without common superscript letters indicate significant difference ($P < 0.01$).
n indicates the number of mice for each treatment.

Table S6-1. PC did not influence relative ATP level in cumulus cells or oocytes in D-gal-induced aging mice

Treatment	Relative ATP level Mean ± SEM (n)	
	cumulus cells	oocytes
Ctrl	1.07 ± 0.14 (8)[a]	1.55 ± 0.09 (8)[a]
D-gal	1.23 ± 0.13 (8)[a]	1.40 ± 0.08 (8)[a]
D-gal+PC	1.33 ± 0.17 (8)[a]	1.41 ± 0.09 (8)[a]

Within column, data without common superscript letters indicate significant difference ($P < 0.05$).
n indicates the number of mice for each treatment.

Table S6-2. Mitochondrial distribution in D-gal-induced oocytes was normalized by PC

Treatment	Mitochondrial distribution (%) Mean ± SEM (n)	
	even	aggregated
Ctrl	94.24 ± 1.39 (6)[a]	5.76 ± 1.39 (6)[a]
D-gal	62.03 ± 6.96 (6)[b]	37.97 ± 6.96 (6)[b]
D-gal+PC	88.95 ± 1.57 (6)[a]	11.05 ± 1.57 (6)[a]

Within column, data without common superscript letters indicate significant difference ($P < 0.001$).
n indicates the number of mice for each treatment.

Table S7. PC reduced high ROS levels induced by D-gal in MII oocytes

Treatment	ROS relative fluorescence Mean ± SEM (*n*)
Ctrl	123.44 ± 10.54 (6)[a]
D-gal	370.14 ± 56.83 (6)[b]
D-gal+PC	181.25 ± 28.22 (6)[ac]

Data without common superscript letters indicate significant difference (ab, $P < 0.001$; bc, $P < 0.01$).
n indicates the number of mice for each treatment.

Table S8. PC inhibited D-gal-induced early apoptosis in MII oocytes

Treatment	Oocytes with early stage apoptosis (%) Mean ± SEM (*n*)
Ctrl	6.19 ± 0.62 (6)[a]
D-gal	19.89 ± 1.07 (6)[b]
D-gal+PC	6.39 ± 1.14 (7)[a]

Data without common superscript letters indicate significant difference ($P < 0.001$).
n indicates the number of mice for each treatment.

Table S9-1. PC rescued litter size in D-gal-treated mice

Treatment	Litter size<br>Mean ± SEM (*N*)
Ctrl	8.69 ± 0.47 (16)[a]
D-gal	6.15 ± 0.73 (20)[b]
D-gal+PC	8.68 ± 0.67 (19)[ac]

Data without common superscript letters indicate significant difference (ab, $P < 0.05$; bc, $P < 0.01$).
N indicates the number of female mice with plugs and that gave birth for each treatment.

Table S9-2. Offspring birth weight was not significantly different among control, D-gal and D-gal+PC groups

Treatment	Birth weight (g)<br>Mean ± SEM (*N*, *n*)
Ctrl	1.57 ± 0.05 (16, 139)[a]
D-gal	1.54 ± 0.06 (20, 123)[a]
D-gal+PC	1.39 ± 0.05 (19, 165)[a]

Data without common superscript letters indicate significant difference ($P < 0.05$).
N indicates the number of female mice with plugs and that gave birth for each treatment. *n* indicates the total number of offspring for each treatment.

Table S9-3. Postnatal growth of female pups from weeks 1 to 8

week	Treatment (*N*, *n*) Mean ± SEM		
	Ctrl (16, 69)	D-gal (19, 62)	D-gal+PC (19, 82)
1	5.28 ± 0.06[a]	5.22 ± 0.06[a]	5.02 ± 0.07[a]
2	8.94 ± 0.06[a]	8.91 ± 0.07[a]	8.37 ± 0.08[a]
3	13.07 ± 0.06[a]	12.78 ± 0.07[a]	12.46 ± 0.05[a]
4	19.03 ± 0.06[a]	18.73 ± 0.06[a]	18.26 ± 0.13[a]
5	21.06 ± 0.06[a]	20.81 ± 0.08[a]	20.42 ± 0.07[a]
6	22.37 ± 0.06[a]	22.26 ± 0.08[a]	21.91 ± 0.16[a]
7	23.36 ± 0.06[a]	23.30 ± 0.08[a]	23.14 ± 0.13[a]
8	24.34 ± 0.06[a]	24.34± 0.08[a]	23.97± 0.18[a]

Within row, data without common superscript letters indicate significant difference ($P < 0.05$).

N indicates the number of female mice with plugs and that gave birth for each treatment. *n* indicates the total number of offspring for each treatment.

Table S9-4. Postnatal growth of male pups from weeks 1 to 8

week	Treatment (*N*, *n*) Mean ± SEM		
	Ctrl (16, 70)	D-gal (18, 61)	D-gal+PC (18, 83)
1	5.21 ± 0.05[a]	5.01 ± 0.07[a]	4.74 ± 0.07[a]
2	11.77 ± 0.09[a]	11.37 ± 0.07[a]	11.51 ± 0.04[a]
3	17.15 ± 0.09[a]	17.51 ± 0.06[a]	17.93 ± 0.07[a]
4	20.98 ± 0.09[a]	21.44 ± 0.06[a]	21.77± 0.07[a]
5	24.71 ± 0.09[a]	25.08 ± 0.06[a]	25.43 ± 0.07[a]
6	27.12 ± 0.09[a]	27.65 ± 0.06[a]	28.05 ± 0.08[a]
7	28.54 ± 0.09[a]	28.91± 0.06[a]	29.10 ± 0.08[a]
8	30.01 ± 0.11[a]	30.19 ± 0.07[a]	30.27 ± 0.08[a]

Within row, data without common superscript letters indicate significant difference ($P < 0.05$).

N indicates the number of female mice with plugs and that gave birth for each treatment. *n* indicates the total number of offspring for each treatment.
